# A radiocarbon bomb pulse model for estimating the age of North Atlantic cetaceans

**DOI:** 10.1098/rsbl.2024.0350

**Published:** 2024-11-27

**Authors:** Eva Garde, Susanne Ditlevsen, Jesper Olsen, Mads Peter Heide-Jørgensen

**Affiliations:** ^1^ Greenland Institute of Natural Resources (GINR), Greenland; ^2^ Department of Mathematical Sciences, University of Copenhagen, Copenhagen, Denmark; ^3^ Department of Physics and Astronomy, Aarhus AMS Centre (AARAMS), Aarhus University, Aarhus, Denmark

**Keywords:** bomb radiocarbon, isotope, age estimation, narwhal, marine mammal, Arctic

## Abstract

The tusk of the male narwhal is a prolonged canine tooth, reaching a size of up to 3 m in length. The tusk erupts through the young narwhal’s upper left lip and, over time, develops into an elongated structure composed of dentine growth layers with an outer coating of cementum. In this study, we utilized bomb radiocarbon (^14^C) to estimate the ages of three narwhal tusks, which allowed us to validate the assumption that one growth layer is deposited annually in narwhal tusks. We used radiocarbon values from samples collected from the annual growth layers along the lengths of the erupted tusks and from the tip and base of embedded teeth, together with published radiocarbon data from three North Atlantic whale species, for the purpose of building a model of the incorporation of bomb radiocarbon in marine mammal tissues. The results obtained contribute significantly to our understanding of narwhal growth and longevity, enhancing our comprehension of isotope flow in the Arctic marine environment and their incorporation into the living tissue of a marine top predator. The bomb radiocarbon model can serve as a valuable reference chronology for dating museum or field specimens of narwhals and other North Atlantic marine animals.

## 1. Introduction

Accurate age estimation techniques are vital for determining key biological parameters, including age at sexual maturity, first reproduction, age structures and life expectancy [1]. These parameters play a pivotal role in population dynamic models of marine mammals, aiding the assessment of sustainable resource utilization and providing guidance on sustainable removals [2]. In Greenland, Inuit hunters rely on narwhals (*Monodon monoceros*) for meat, dog food and tusks for crafts [3]. Narwhal skin, or ‘mattak’, holds cultural and commercial value, making hunting a key income source and narwhal population management a contentious issue [4,5]. This emphasizes the need to develop and validate narwhal age estimation methods.

Age determination of odontocete (toothed) whales traditionally involves counting of dentine growth layer groups (GLGs) consisting of an opaque and a translucent layer deposited annually [6–8]. The erupted tusk of the male narwhal is composed of dentine GLGs with an exterior coating of cementum, and it likely continues to grow throughout the male narwhal’s life. The layers are considered to be deposited annually [9], a phenomenon observed in the teeth of other marine mammals [10,11], including the narwhal’s closest relative, the beluga whale (*Delphinapterus leucas*; [12]), although not yet directly proven for narwhals. Besides providing a mean of estimating age, the GLGs also capture materials such as trace elements and isotopes in the dentine growth layers, reflecting the narwhal’s diet and habitat [13,14]. Given that narwhals are long-lived creatures (greater than 80 years; [15]), the tusk effectively serves as a chronological time series archive of information about the narwhal and its environment throughout its lifetime [16]. In female narwhals, a tusk rarely erupts; instead, they possess two small embedded teeth in the upper jaw, which, similarly to the erupted tusk, store valuable information [17]. Males possess one erupted tusk and one embedded tooth. The embedded teeth cease growing at some point in the life of a narwhal, and they, therefore, only provide a partial chronology of the narwhal’s life [9].

Another well-established and dependable method used in the validation of age determination techniques of marine organisms is ‘bomb’ radiocarbon dating [18–20]. In hunted populations of cetaceans, where biological material can be sampled through collaborations with local hunters, the method has been used for age validation of beluga whales using teeth [12], humpback whales (*Megaptera novaeangliae*) using eye lenses [21] and, recently, fin whales (*Balaenoptera physalus*) using earplugs [22]. The method represents one of the most robust age-validation approaches available for long-lived organisms that develop growth bands [23]. The sharp increase in atmospheric radiocarbon content resulting from nuclear testing in the mid- to late-1950s, known as the ‘bomb pulse’, rapidly permeated food webs worldwide, including marine systems in the North Atlantic and Arctic Oceans. This isotopic shift was incorporated into all organisms growing during that time, and ^14^C has since then been applied as a chemical baseline marker [24]. In this study, we investigated the use of bomb radiocarbon as a marker incorporated into discrete growth layers of three narwhal tusks to determine if the individual narwhals were born before or after the initial occurrence of the bomb pulse in the North Atlantic in 1958 [18]. We used the isotopes of δ^13^C and δ^15^N to demonstrate that the carbon source is of dietary origin and that the prenatal dentine isotopically can be distinguished from the postnatal dentine in the tusks and embedded teeth. Based on our findings, we re-evaluate previously published age estimates of the three tusks and investigate the assumption that one dentine growth layer forms annually in narwhal tusks. Using the radiocarbon data from the individual growth layers sampled along the length of the erupted narwhal tusks and from the tip and base of embedded teeth along with published radiocarbon values from three additional whale species, we establish a bomb radiocarbon model for Arctic and North Atlantic marine mammals for future dating purposes.

## 2. Material and methods

### (a) Sample collection and temporal timelines in tusks and teeth

**Figure 1.**
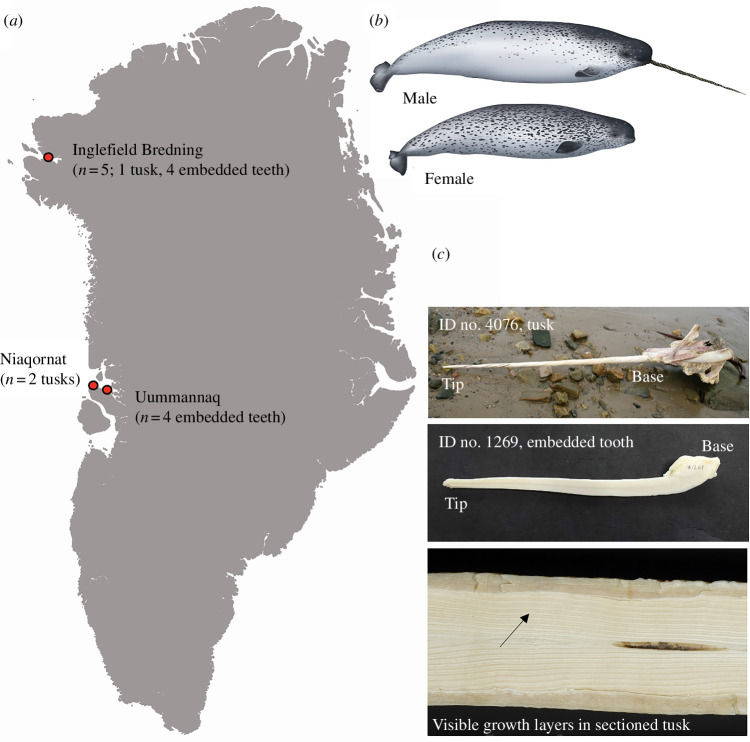
Sampling sites in Greenland and examples of an erupted tusk and an embedded tooth used in this study. (*a*) Map of Greenland showing sampling sites and number of specimens collected. (*b*) Illustration of a male narwhal with an erupted tusk and a female with no tusk. (*c*) Tip and base of tusk no. 4076; sectioned embedded tooth no. 1269; piece of sectioned tusk no. 4076, arrow pointing at growth layers.

Narwhal tusks (*n* = 3; males) and embedded teeth (*n* = 8; six females and two males) were obtained from the Inuit hunt of narwhals in Northwest Greenland in collaboration with local hunters (figure 1*a–c*). Collection years were spanning from 1984 to 2008, and the year of death was recorded for all specimens (table 1). The three tusks had previously been sectioned and age estimated by counting of GLGs (table 1 and figure 1*c*; [26]). Two of the three tusks (no. 4976 and no. 956) were, based on length and number of GLGs, assumed to belong to narwhals born prior to the onset of the bomb pulse in the North Atlantic. The eight teeth were sourced from narwhals with known year of death post the bomb pulse. They were assumed to have been born before the bomb pulse, which was assessed based on body measurements (*n* = 7), and from age estimates obtained from the counting of GLGs in this study (*n* = 8) and/or from published age estimates using the aspartic acid racemization technique (AAR; *n* = 4; table 1; [25]).

**Table 1.** Narwhal tusks and teeth used in this study with information on specimen ID, year of death, sampling site (IB: Inglefield Bredning; NI: Niaqornat; UU: Uummannaq), sex of the whale, specimen type, length (cm) and, if the neonatal line was present, body length (cm) of the whale, number of ^14^C samples collected, number of growth layers estimated and the aspartic acid racemization ages (AAR) in years [25].

specimen ID	year of death	sampling site in Greenland	sex	specimen	neonatal line present	length of specimen (cm)	body length (cm)	^14^C samples (n)	growth layers (*n*)	AAR ages (years; ± s.e.)
4076	2008	IB	M	tusk	—	255	491	17	60^a^	—
956	2010	NI	M	tusk	—	248	440	10	70^a^	—
953	2008	NI	M	tusk	(+)	201	471	3	26	—
1336	1985	IB	M	tooth	+	230	450	2	20	—
1338	1985	IB	M	tooth	+	197	490	2	16	—
560	1993	UU	F	tooth	+	19.3	410	2	10	67.9 (4.9)
537	1993	UU	F	tooth	+	17.5	400	2	11	47.8 (3.6)
562	1993	UU	F	tooth	+	20.2	425	2	10	101.0 (7.2)
559	1993	UU	F	tooth	+	17.0	390	2	10	63.5 (4.6)
1269	1984	IB	F	tooth	+	20.4	—	2	10	—
1229	1984	IB	F	tooth	+	19.9	330	2	9	—

^a^ Estimated number of growth layers missing in the tip: no. 4076, *n* = 10; no. 956, *n* = 1−3 [26].

The tusks and teeth increments represent periods in time. Where the very tip represents the oldest part, formed during the fetal stage and distinguished by the presence of the neonatal line [9], thus portraying the unborn animal, the base is the most recently deposited part, representing the older narwhal (figure 1*c*). When the tip is worn or broken off the tusk [27], the first available growth layer will represent the postnatal or young narwhal. The age represented by this first layer depends on the number of layers that are missing. For tusks no. 4076 and no. 956, the tip was broken off, and the neonatal line was not present. For tusk no. 953, the tip was worn but complete, and it was uncertain if the neonatal line was present (table 1). The growth layers from tip to base represent a chronological timeline from the year of birth to the year the last growth layer was formed.

### (b) Sample preparation and isotope analyses

Dentine powder samples were obtained by drilling into the individual GLGs of the erupted tusks and the tip and base of the embedded teeth for radiocarbon (^14^C; *n* = 46) and stable isotope (δ^13^C and δ^15^N; *n* = 36) analyses using a Foredom K.1030 Portable Rotary Micromotor Kit Portable (table 2). The growth layers at the base of the embedded teeth were very narrow. To obtain sufficient material, these samples cover a few GLGs, representing the final years of the narwhal’s life or the last years before tooth growth cessation.

**Table 2.** Estimated tusk and embedded tooth chronologies and measured stable isotope values for the narwhals. The table includes information on specimen ID, the estimated chronology of sampled growth layers and the year when the growth layer was deposited, the ^14^C (pMC), δ^13^C (‰) and δ^15^N (‰) values with associated standard deviations ( ± s.d.) and the laboratory ID.

specimen ID	growth layer	year of growth layer deposition	^14^C (pMC)	δ^13^C (‰)	δ^15^N (‰)	laboratory ID
4076^a^	11	1960	98.28 ± 0.51	—	—	—
4076^a^	12	1961	100.69 ± 0.38	—	—	—
4076^a^	11	1960	99.55 ± 0.40	—	—	—
4076^a^	14	1963	100.92 ± 0.38	—	—	—
4076^a^	22	1971	103.31 ± 0.49	—	—	—
4076^a^	33	1982	104.26 ± 0.39	—	—	—
4076^a^	48	1997	102.36 ± 0.39	—	—	—
4076^b^	11	1960	99.03 ± 0.34	−14.5 ± 0.2	17.5 ± 0.4	AAR-31974
4076^b^	12	1961	99.96 ± 0.30	−14.9 ± 0.2	17.6 ± 0.4	AAR-31975
4076^b^	13	1962	100.52 ± 0.28	−14.5 ± 0.2	17.2 ± 0.2	AAR-31976
4076^b^	17	1966	99.84 ± 0.27	−14.7 ± 0.2	17.6 ± 0.4	AAR-31977
4076^b^	20	1969	101.21 ± 0.31	−14.2 ± 0.2	18.0 ± 0.4	AAR-31978
4076^b^	21	1970	101.02 ± 0.32	−13.9 ± 0.2	18.8 ± 0.2	AAR-31979
4076^b^	22	1971	101.78 ± 0.29	−13.7 ± 0.2	18.1 ± 0.4	AAR-31980
4076^b^	32	1981	103.62 ± 0.31	−13.7 ± 0.2	18.7 ± 0.4	AAR-31981
4076^b^	44	1993	103.60 ± 0.29	−13.8 ± 0.2	19.5 ± 0.4	AAR-31982
4076^b^	54	2003	103.15 ± 0.34	−13.9 ± 0.2	19.3 ± 0.4	AAR-31983
956^b^	1	1958	94.87 ± 0.26	−14.2 ± 0.2	17.4 ± 0.4	AAR-31984
956^b^	2	1959	96.84 ± 0.28	−14.1 ± 0.2	17.2 ± 0.2	AAR-31985
956^b^	3	1960	98.33 ± 0.28	−14.2 ± 0.2	16.8 ± 0.4	AAR-31986
956^b^	5	1962	100.21 ± 0.27	−14.2 ± 0.2	16.6 ± 0.4	AAR-31987
956^b^	7	1964	100.54 ± 0.28	−14.3 ± 0.2	16.4 ± 0.4	AAR-31988
956^b^	9	1966	98.61 ± 0.31	−14.0 ± 0.2	17.0 ± 0.4	AAR-31989
956^b^	12	1969	100.29 ± 0.31	−14.4 ± 0.2	16.8 ± 0.4	AAR-31990
956^b^	23	1980	102.91 ± 0.27	−14.2 ± 0.2	16.8 ± 0.4	AAR-31991
956^b^	35	1992	103.69 ± 0.29	−14.1 ± 0.2	18.6 ± 0.2	AAR-31992
956^b^	45	2002	103.37 ± 0.29	−14.5 ± 0.2	17.6 ± 0.4	AAR-31993
953^a^	1	1982	106.01 ± 0.40	—	—	—
953^a^	6	1987	105.49 ± 0.40	—	—	—
953^a^	16	1997	103.84 ± 0.44	—	—	—
1269_tip^a^	0	1958	95.57 ± 0.30	−14.5 ± 0.2	21.0 ± 0.1	AAR-35621
1269_base^a^	10	1968	101.28 ± 0.36	−14.9 ± 0.2	17.4 ± 0.1	AAR-35622
1229_tip^a^	0	1975	101.31 ± 0.38	−14.7 ± 0.2	19.8 ± 0.1	AAR-35623
1229_base^a^	9	1984	103.16 ± 0.34	−14.7 ± 0.1	17.6 ± 0.1	AAR-35624
1336_tip^a^	0	1938	92.65 ± 0.39	−14.8 ± 0.2	20.1 ± 0.1	AAR-35625
1336_base^a^	20	1958	94.12 ± 0.38	−14.6 ± 0.2	17.7 ± 0.1	AAR-35626
1338_tip^a^	0	1942	92.68 ± 0.33	−14.0 ± 0.2	20.3 ± 0.1	AAR-35627
1338_base^a^	16	1958	94.32 ± 0.57	−14.5 ± 0.2	17.8 ± 0.1	AAR-35628
560_tip^a^	0	1925	92.66 ± 0.36	−14.7 ± 0.1	20.7 ± 0.1	AAR-35629
560_base^a^	10	1935	91.81 ± 0.38	−14.8 ± 0.2	18.4 ± 0.1	AAR-35630
537_tip^a^	0	1945	92.47 ± 0.30	−15.3 ± 0.2	20.0 ± 0.1	AAR-35631
537_base^a^	11	1956	93.26 ± 0.36	−14.3 ± 0.2	18.3 ± 0.1	AAR-35632
562_tip^a^	0	1892	93.27 ± 0.35	−14.4 ± 0.2	20.5 ± 0.1	AAR-35633
562_base^a^	10	1902	92.92 ± 0.34	−14.1 ± 0.2	18.3 ± 0.1	AAR-35634
559_tip^a^	0	1930	93.36 ± 0.32	−14.4 ± 0.2	20.3 ± 0.1	AAR-35635
559_base^a^	10	1940	93.20 ± 0.35	−14.4 ± 0.1	17.9 ± 0.1	AAR-35636

^a^ Center for Accelerator Mass Spectrometry, Lawrence Liverpool National Laboratory, USA (*n* = 10).

^b^ The Aarhus AMS Centre, Aarhus University, Denmark (*n* = 36).

The samples were analysed at the Aarhus AMS Centre (AARAMS, *n* = 36) and the Center for Accelerator Mass Spectrometry, Lawrence Livermore National Laboratory (*n* = 10). Collagen was extracted using the modified Longin procedure with ultrafiltration [28–30]. The samples were dissolved in hydrochloric acid at 4°C for several days. The acid was renewed regularly until the mineral fraction was removed completely. Humic substances were removed using 0.2 M NaOH at 4°C, with frequent changes of the NaOH, each step lasting several hours, until the solution stayed clear. The samples were subsequently rinsed with 0.01 M HCl and gelatinized in 0.01 M HCl at 58°C for 3−4 days. The collagen was subsequently converted to CO_2_ by combustion in sealed evacuated quartz tubes with 200 mg CuO. The CO_2_ was reduced to graphite by the H_2_ reduction method using an iron catalyst and MgClO_4_ to remove the water [31,32]. The samples were ^14^C dated using the HVE 1 MV tandetron accelerator AMS system at AARAMS [33]. ^14^C dates are reported as per cent modern carbon (pMC) normalized to −25‰ according to the international convention using the ^13^C/^12^C ratios from the AMS analysis [34]. Stable isotopes δ^13^C and δ^15^N were measured on the extracted collagen using a continuous-flow IsoPrime IRMS coupled to an Elementar PyroCube elemental analyser at AARAMS. An in-house standard Gel-A was used as primary standard, yielding ± 0.2‰ and ± 0.3‰ for carbon and nitrogen analysis, respectively. Further, secondary in-house and international standards were used to check the normalization to the VPDB and AIR scale.

Samples from the tip, or the first available growth layer in the absence of the tip, were used as radiocarbon reference points to infer if the narwhal was born prior to or after the onset of the bomb pulse in 1958. Dentine formed *before* the onset of the bomb pulse in the North Atlantic would contain only natural, low-level ^14^C concentrations of about 95 pMC; *during* the transition period, intermediate and increasing ^14^C levels; or *after* elevated levels above 95 pMC [23]. Thus, we used a pMC value of 95 to serve as the threshold for distinguishing growth layers formed prior to (less than 95 pMC) or after (greater than 95 pMC) the bomb pulse, as applied in the case of the Greenland shark (*Somniosus microcephalus*) [20]. To confirm our narwhal age chronologies, we compared the ^14^C levels in narwhal dentine with published ^14^C reference chronologies from the same region and from a surface ocean mixed-layer bomb pulse model [18,20,35].

### (c) Bomb radiocarbon model

We developed a model reflecting bomb radiocarbon in Arctic and North Atlantic marine mammals using data from this study (table 2) and integrating published ^14^C values from beluga whales [12], humpback whales [21] and fin whales [22]. To our knowledge, this is the only published data on incremental and temporal radiocarbon deposition for North Atlantic cetaceans covering the bomb pulse period.

A model for the time evolution of ^14^C in whale tissue around the bomb pulse provides a tool for dating measurements from other individuals and for conducting sanity checks on different dating methods. To describe the time-varying pMC values caused by the bomb pulse, we developed a five-parameter model relating pMC to year via a function as follows:


$$pMC\left(year\right)=a+b\;\frac{exp\;\left(\left(c-year\right)/d_{2}\right)}{1+exp\;\left(\left(c-year\right)/d_{1}\right)}.$$

This model assumes that before the bomb pulse, the pMC level is approximately equal to *a*. This is probably not entirely true; however, there are few data points before the bomb pulse, and no trend was detected in the data. The bomb pulse causes the pMC level to rise at a speed determined by the time parameter $d_{1}$. We expect that after reaching a peak some years after the bomb pulse, the surface ^14^C level will decrease due to mixing with the ^14^C depleted deep ocean, determined by the time parameter $d_{2}$, at a much slower speed than the rise, so that $d_{2}\gg d_{1}$. Parameter *c* is the year at which the pMC takes the value a+b/2 and is expected to lie a few years after the increasing surface ocean ^14^C levels caused by the atmospheric nuclear bomb tests. The height of the peak is determined by b and the timescale separation. It takes the value $a+b{{(d_{1}/d_{2})(d}_{2}/d_{1}-1)}^{1-d_{1}/d_{2}}$ and occurs in the year ${c+d_{1}log(d}_{2}/d_{1}-1)$.

Parameters were estimated with weighted nonlinear least squares (nls in R v. 4.2.1). Pointwise 95% confidence interval around the regression line was calculated from 1000 bootstrap samples based on residuals (boot within package boot 1.3–28 in R). An 80% prediction interval was constructed by the 10% and 90% quantiles from 1000 simulated samples from the fitted model. To test the accuracy of the model, we conducted an out-of-sample analysis by leave-one-out cross-validation (see electronic supplementary materials for details).

## 3. Results and discussion

### (a) Carbon and protein sources

We used the stable isotopes δ^13^C and δ^15^N to evaluate the isotope source in the dentine samples. Depleted ^13^C and enriched ^15^N levels are indicative of metabolic and dietary carbon and signify a high trophic level, a characteristic shown in various marine mammals [12,14,36–38]. We found that the narwhal tusks and embedded teeth showed a depletion in ^13^C, with δ^13^C values ranging from −13.7‰ to −15.3‰. The average δ^13^C value was −14.4‰ (*n* = 36, table 2; electronic supplementary material, figure S2), which closely aligns with the average δ^13^C value of −14.3‰ reported by Stewart *et al*. [12] for beluga teeth. These depleted values demonstrate that the carbon source is of dietary origin.

Prenatal dentine of narwhals can be distinguished from postnatal dentine by the δ^15^N level [38]. Zhao *et al*. [38, fig. 3] found that δ^15^N was considerably higher in the prenatal dentine of narwhals, compared to postnatal dentine, suggesting that the mother’s protein sources support fetal growth. Elevated δ^15^N levels were similarly observed at the prenatal tip of the embedded narwhal teeth (average: 20.3‰) compared to the postnatal base (average: 17.9‰, table 2; electronic supplementary material, figure S2). We propose that this feature can be used to assess the presence of prenatal dentine in cases where the presence of the neonatal line is uncertain. The tusks of no. 956 and no. 4076 were incomplete, lacking the neonatal line and growth layers at the tip, and it was previously estimated that 1−3 and 10 GLGs were missing, respectively (table 1; [26]). This observation is further supported by δ^15^N values from the first available layer at the tip of 17.4‰ ± 0.4 s.d. and 17.5‰ ± 0.4 s.d., respectively, which fall within the same range as the postnatal δ^15^N values of the eight embedded teeth (table 2; electronic supplementary material, figure S2). The δ^15^N levels exhibited a temporal increasing trend in both no. 956 and no. 4076, perhaps due to an age-related dietary shift. However, further research is needed to better understand individual and age-related foraging behaviours in relation to isotope profiles. No stable isotope values were generated for tusk no. 953.

### (b) Age estimation of the three tusks from radiocarbon

**Figure 2.**
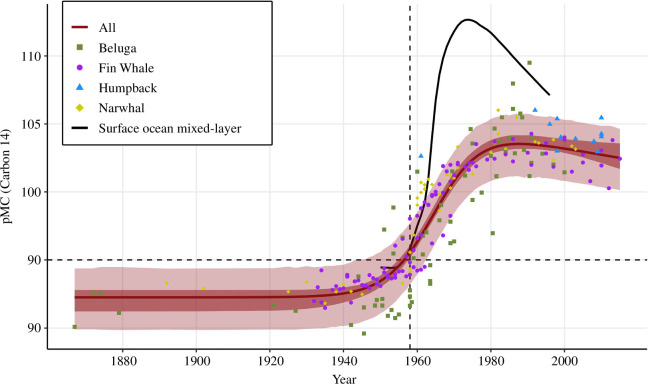
Radiocarbon values (pMC) against year for narwhals (*n* = 46, yellow diamonds, this study), belugas (*n* = 68, green squares, [26]), humpback whales (*n* = 12, blue triangles, [21]) and fin whales (*n* = 96, purple circles, [22]). The dark red line is the model fitted to radiocarbon values from all four whale species. The dark-shaded area shows the 95% confidence intervals, and the light-shaded area indicates the 80% prediction intervals. The year of maximum radiocarbon is estimated to 1988 with a pMC value of 103.5. The solid black line shows a modelled surface ocean mixed-layer bomb pulse for comparison [18,35]. The dotted lines mark the bomb pulse onset in 1958 and the 95 pMC threshold, distinguishing growth layers deposited before (less than 95 pMC) or after (greater than 95 pMC) the bomb pulse.

A ^14^C value of 94.87 ± 0.26 pMC from the first available growth layer from tusk no. 956 indicated that its deposition happened just around the time of the occurrence of the bomb pulse in the North Atlantic, using the 95 pMC criteria. Subsequent growth layers from no. 956 displayed an abrupt increase in ^14^C values, followed by a levelling of (figure 2; electronic supplementary material, figure S1). This sharp rise in ^14^C values aligns with the patterns observed in the North Atlantic reference chronologies from mid-1950s to the start of 1960s, followed by a decrease in ^14^C pMC from approximately 1970 and onward [18]. Based on this, we conclude that the first available layer of tusk no. 956 was formed in 1958, the first year when the bomb pulse was registered in other chronologies from the North Atlantic [18]. Accounting for 1−3 missing layers in the tip, narwhal no. 956 had a maximum age of 55 years, when it died in 2010.

The ^14^C values from 17 growth layers of tusk no. 4076 ranged from 98.28 ± 0.51 to 104.26 ± 0.39 pMC (table 2), exhibiting a sudden rise followed by stabilizing, as also observed for tusk no. 956 (figure 2; electronic supplementary material, figure S1). Given the absence of 10 GLGs from the tip [26], GLG 11 was the first available to be sampled, showing a ^14^C value of 98.28 ± 0.51 pMC. A similar pMC value for narwhal no. 956 was found in the year 1960 (98.33 ± 0.28; table 2). If we assume that narwhal tusks display similar ^14^C values at specific years in time and take into account the 10 missing GLGs, narwhal no. 4076 would have been born around 1950. When it died in 2008, it was then approximately 58 years. Narwhals no. 4076 and no. 956 were both large, adult males with extensive tusks (table 1), and we, therefore, anticipate similar ages, as also suggested by the radiocarbon values. Comparison of the ^14^C ages estimated in this study with ages based on the number of GLGs, assuming deposition of one GLG^-yr^, reported in a previous study [26], show good agreement for no. 4076 (58 versus 60 years, respectively), whereas the age based on number of GLGs for no. 956 seems to have been overestimated (55 versus 70, respectively). It is important to note that GLGs in tusks from older animals are often more challenging to discern and count due to compact layering, accessory layers, wear of the tusks and frequently broken tips [28,38,39]. Also, there is increased uncertainty in the GLG counts in the absence of the neonatal line, which is essential for reliable age estimates based on GLG counts [3]. We consider the ^14^C age of 55 years for no. 956 as more accurate than the previous estimate derived from the number of GLGs. For no. 953, the elevated but declining ^14^C values align with the reported number of GLGs (*n* = 26, [26]) and a birth year of 1982, assuming an annual deposition rate, when compared with reference chronologies.

### (c) Model of bomb radiocarbon in North Atlantic whales

We fitted the model to the data from the narwhals, belugas and fin whales separately, as well as for all four species combined (table 3 and figure 2). The data from narwhals did not converge, and they were instead fitted to a sigmoidal model with no decay after the bomb pulse. For humpback whales, only measurements after the bomb were available, but these data had a marginal impact on the overall model estimation (results not shown). The leave-one-out age prediction errors for individuals with at least one pMC value between 95 pMC and the max fitted value had a mean of 0.72 years, a median of 1.56 years and a standard error of 7.01 years. The radiocarbon model presented in this study, thus, offers a promising tool for dating whale samples from across the North Atlantic, utilizing the parameters outlined in table 3 (for details, see electronic supplementary material, table S1).

**Table 3.** Model estimates for narwhal, beluga and fin whale and all whale species combined.

parameter	species	estimate	lower	upper
*a*, initial level before bomb pulse	narwhal	92.6	91.6	93.5
*a*, initial level before bomb pulse	fin whale	92.9	92.3	93.5
*a*, initial level before bomb pulse	beluga	91.1	89.2	93.0
*a*, initial level before bomb pulse	all	92.2	91.5	93.0
*b*, asymptotic level after bomb (in four-parameter model, compare to *b-a* in five-parameter model)	narwhal	103.3	102.6	104.1
*b*, max increment during bomb pulse	fin whale	11.0	8.8	13.1
*b*, max increment during bomb pulse	beluga	25.4	14.4	36.4
*b*, max increment during bomb pulse	all	12.9	10.2	15.6
*c*, approximate year at half rise of bomb pulse^a^	narwhal	1960	1959	1962
*c*, approximate year at half rise of bomb pulse^a^	fin whale	1964	1962	1966
*c*, approximate year at half rise of bomb pulse^a^	Beluga	1983	1965	2001
*c*, approximate year at half rise of bomb pulse^a^	all	1965	1962	1968
$d_{1}$, time parameter of bomb pulse	narwhal	4.5	2.8	6.2
$d_{1}$, time parameter of bomb pulse	fin whale	5.0	3.5	6.4
$d_{1}$, time parameter of bomb pulse	beluga	7.8	5.4	10.2
$d_{1}$, time parameter of bomb pulse	all	6.4	4.8	8.2
$d_{2}$, time parameter of slow decay after bomb pulse	narwhal	—	—	—
$d_{2}$, time parameter of slow decay after bomb pulse	fin whale	339.6	−231.3	910.6
$d_{2}$, time parameter of slow decay after bomb pulse	beluga	21.9	−10.8	54.5
$d_{2}$, time parameter of slow decay after bomb pulse	all	218.2	−47.0	483.4

^a^ Year where pMC = *a* + *b*/2.

The initial appearance of bomb radiocarbon in different biological materials from our four whale species reflects the patterns detected in published marine reference chronologies worldwide and across a variety of species and tissues [18,20–22,26]. Since the carbon source for the four whale species is derived from their diet (e.g. fish and crustaceans), feeding at higher trophic levels or on long-lived prey would potentially slow the rate of increase in the bomb radiocarbon signal, extending its presence over a longer period [12]. Stewart *et al*. [12] concluded that this effect was observed in the belugas, which feed at several trophic levels. In belugas, the radiocarbon signal initially appeared at the expected year, but the subsequent incorporation extended beyond the 1970s into the 1980s. This trend was not observed in the other whale species, including narwhals, which also occasionally feed on long-lived species, e.g. Greenland halibut (*Reinhardtius hippoglossoides*; table 3 and figure 2; electronic supplementary material, figure S1). However, age estimation in belugas may be more prone to errors due to the difficulty in precisely enumerating GLGs, which in turn affects the interpretation of bomb pulse trajectory [7].

## 4. Conclusion

This study provides compelling evidence that narwhal dentine growth layers serve as annual archives with information about the individual’s habitat. This information can be extracted and used as the basis for constructing chronological time series for an Arctic marine mammal and its ecosystems. Analysis of narwhal tusks, embedded teeth or similar biological incremental materials that preserve chronological records of biogeochemical data holds the potential to yield profound insights into fundamental questions related to age, life history, habitat, environmental fluctuations and climate change. The adoption of new methods and innovative technologies for analysing and interpreting data on ^14^C and stable isotopes represents a promising avenue for delving into the past and gaining a deeper understanding of the present. Furthermore, the radiocarbon model established in this study holds valuable utility, serving as a reference for dating carbon-based samples throughout the entire North Atlantic region. This resource becomes particularly significant when considering archived collections in museums, enhancing our capacity to uncover historical insights, or for obtaining age structures and other age-dependent parameters used in population modelling and management of hunted species.

## Data Availability

Information on the narwhal specimens and raw data used in this study is enclosed in tables 1 and 2. Model equations are shown in the manuscript and the electronic supplementary material, and model parameters are listed in table 3. Importation of narwhal specimens into Denmark was authorized by CITES permits IM 0721-199/08, IM 0721-200/08 and DK-2021-0067486-01. The specimens are currently stored at the GINR in Copenhagen, Denmark. For information and access to specimens, contact lead author Senior Scientist Eva Garde. Supplementary material is available online [40].
